# Comparison and Performance Validation of Calculated and Established Anaerobic Lactate Thresholds in Running

**DOI:** 10.3390/medicina57101117

**Published:** 2021-10-16

**Authors:** Sanghyeon Ji, Aldo Sommer, Wilhelm Bloch, Patrick Wahl

**Affiliations:** 1 The German Research Centre of Elite Sport, German Sport University Cologne, 50933 Cologne, Germany; hyeon7748@gmail.com (S.J.); a.sommer@dshs-koeln.de (A.S.); w.bloch@dshs-koeln.de (W.B.); 2 Department of Sports Medicine and Exercise Physiology, Institute of Sport Sciences, Goethe University Frankfurt, 60487 Frankfurt, Germany; 3 Department of Molecular and Cellular Sport Medicine, Institute of Cardiology and Sports Medicine, German Sport University Cologne, 50933 Cologne, Germany; 4 Institute of Interdisciplinary Exercise Science and Sports Medicine, Medical School Hamburg, 20457 Hamburg, Germany

**Keywords:** aerobic capacity, anaerobic capacity, maximal lactate production rate, exercise testing, endurance performance, metabolism

## Abstract

*Background and Objectives*: This study aimed to compare the calculated running velocity at the anaerobic lactate threshold (cLT_An_), determined by a mathematical model for metabolic simulation, with two established threshold concepts (onset of blood lactate accumulation (OBLA; 4 mmol∙L^−1^) and modified maximal deviation method (mDmax)). Additionally, all threshold concepts were correlated with performance in different endurance running events. *Materials and Methods*: Ten sub-elite runners performed a 30 s sprint test on a cycle ergometer adjusted to an isokinetic mode set to a cadence of 120 rpm to determine maximal lactate production rate (VLa_max_), and a graded exercise test on a treadmill to determine maximal oxygen uptake (VO_2max_). Running velocities at OBLA, mDmax, and cLT_An_ were then compared with each other, and further correlated with running performance over various distances (3000 m, 5000 m, and 10,000 m). *Results*: The mean difference in cLT_An_ was −0.13 ± 0.43 m∙s^−1^ and −0.32 ± 0.39 m∙s^−1^ compared to mDmax (*p* = 0.49) and OBLA (*p* < 0.01), respectively. cLT_An_ indicated moderate to good concordance with the established threshold concepts (mDmax: ICC = 0.87, OBLA: ICC = 0.74). In comparison with other threshold concepts, cLT_An_ exhibited comparable correlations with the assessed running performances (cLT_An_: *r* = 0.61–0.76, mDmax: *r* = 0.69–0.79, OBLA: *r* = 0.56–0.69). *Conclusion*: Our data show that cLT_An_ can be applied for determining endurance performance during running. Due to the consideration of individual physiological profiles, cLT_An_ offers a physiologically justified approach to assess an athlete’s endurance performance.

## 1. Introduction

Determination of the blood lactate response during exercise is among the most widely used performance diagnostic tools [1,2]. Blood lactate concentration increases above the resting value with increasing exercise intensity. However, as long as exercise is performed at a constant exercise intensity under a certain intensity threshold, blood lactate concentration remains constant, physiologically known as a steady-state condition [3,4]. At a certain exercise intensity, a minor increment in the workload induces an accelerated blood lactate accumulation and subsequent fatigue-related metabolic consequences, such as the negative impact of hydrogen ion accumulation (acidosis) on muscle function and performance [4,5,6,7]. This considerable point has been defined as the anaerobic lactate threshold (LT_An_), which is generally considered to be a good indicator of individual aerobic endurance performance and can be used for prescribing endurance training intensities [8,9].

In recent decades, researchers have developed several concepts to determine LT_An_. Most LT_An_ concepts are usually applied to lactate performance curves derived from graded incremental exercise tests [8]. Most existing LT_An_ concepts use either fixed lactate concentrations [4,10] or inflection points [11,12] as their determination criteria. However, these criteria are derived either arbitrarily or empirically from the graphical analysis of the lactate performance curve. Moreover, LT_An_ has shown to be strongly dependent on the applied test protocol [13,14] and on the athlete’s training status [15], which is critical because there is no clear standardized test procedure defined, which thus hinders accurate data interpretation and comparison. Therefore, the physiological background and the validity/reliability/comparability of these LT_An_ concepts have been questioned [8].

Lactate production and removal are ongoing processes, which are closely related to metabolic rate but not necessarily to oxygen delivery [5,6,16,17]. There is a continual exchange of lactate between various organs and cells, which can be used as an energy source for oxidative energy production and/or as a major precursor to gluconeogenesis [5,17]. This emphasizes the complexity of metabolic processes behind blood lactate concentrations during exercise or other conditions. Limiting interpretation solely to blood lactate kinetics in response to graded exercise tests allows only scarce insight into the complex metabolic processes of total energy production [18,19].

**Figure 1:**
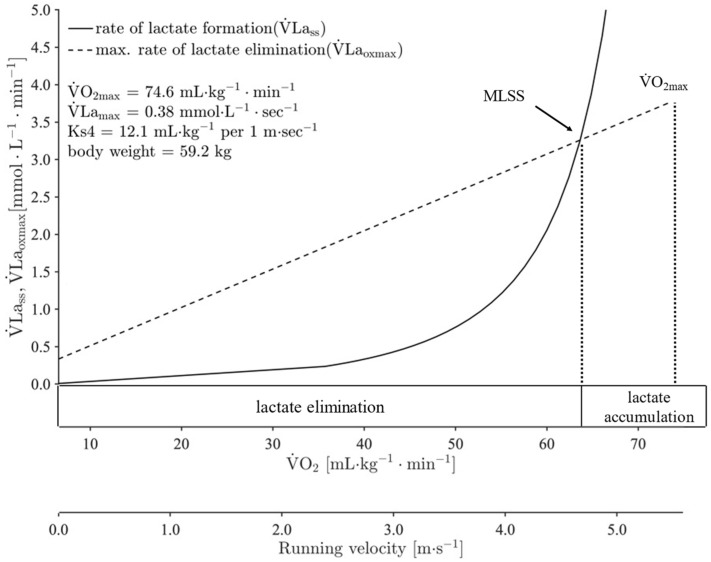
An exemplary description of the mathematical model for metabolic simulation, presenting the gross lactate formation (VLa_ss_) and the maximal lactate elimination rate (VLa_oxmax_) depending on exercise intensity [22]. Maximal lactate steady state (MLSS) is defined as the exercise intensity at which the lactate formation is exactly equal to elimination. VO_2max_ = maximal oxygen uptake; VLa_max_ = maximal lactate production rate; *Ks*4 = individual constant value of the relationship between oxygen demand and running velocity.

In 1984, Mader [20] suggested that the lactate performance curve and the corresponding exercise intensity at LT_An_ may be influenced by aerobic (maximal oxygen uptake; VO_2max_) or anaerobic (glycolytic) capacity (maximal lactate production rate; VLa_max_) separately [20]. Further research confirmed this assumption and showed that different combinations of VO_2max_ and VLa_max_ can result in two identical lactate performance curves with equal LT_An_ [18]. In a more differentiated approach, Mader and Heck [3] proposed a mathematical simulation model of energy production processes in skeletal muscle. Using Michaelis–Menten kinetics, these researchers described the activation of glycolysis as a lactate production system and the oxidative phosphorylation as a combustion system, both depending on the total metabolic rate [3]. Based on this theoretical construct, the term “maximal steady-state of blood lactate (MLSS)” was introduced (as another concept of LT_An_), at which the extent of lactate formation by glycolysis is exactly equal to the maximal elimination rate of lactate by combustion. Thus, no lactate accumulation in blood lactate over time occurs (Figure 1) [3]. Thereby, it was suggested that accelerated accumulation of blood lactate during exercise is due to the saturation of the combustion system (oxidative phosphorylation) [3], which was later verified by subsequent investigations of lactate kinetics during exercise [6,21]. As this mathematical model considers both the maximal aerobic and anaerobic capacities for the determination of LT_An_, it provides differentiated information about the energetic background of LT_An_, as well as the physiological profile of an athlete [18].

Based on Mader’s approach, Hauser et al. [22] applied the mathematical model to calculate the power output at MLSS during cycling using individual VO_2max_- and VLa_max_-values and demonstrated a significant correlation with the experimental determined MLSS, and high reliability in the estimation of MLSS [23]. However, there is a lack of knowledge regarding the transferability of the model to running. Furthermore, the calculation method in the previous study [22] has only been compared to the empirically determined MLSS, but not to the actual athlete’s competition performance, which is an essential aspect for a practical application of a laboratory testing parameter [8]. Therefore, this study aimed to calculate running velocity at LT_An_ using individual VO_2max_ and VLa_max_ and an adapted mathematical method initially described by Mader and Heck [3] and Hauser et al. [22]. The calculated LT_An_ (cLT_An_) was then compared with other established experimentally determined LT_An_ concepts. Additionally, we aimed to validate cLT_An_ against the athlete’s recent performance in endurance running events.

## 2. Materials and Methods

### 2.1. Subjects

Ten sub-elite male middle- and long-distance runners (age = 19.2 ± 3.5 years, body mass = 65.8 ± 5.8 kg, height = 181.7 ± 5.2 cm, VO_2max_ = 69.8 ± 6.7 mL∙kg^−1^ min^−1^, VLa_max_ = 0.39 ± 0.09 mmol L^−1^ s^−1^) participated in this study. Prior to signing the written informed consent of the investigation, all participants were informed about the experimental procedures. The protocols used in this investigation were approved by the Ethics Committee of the university and are in line with the Declaration of Helsinki.

### 2.2. Design

The present investigation consisted of two different performance tests completed on a single day. The body mass was measured before the performance testing (Tanita Corp., Tokyo, Japan). Participants were instructed to arrive in the laboratory in a rested, 2 h postprandial, and well-hydrated state. They were ordered to avoid strenuous exercise for at least 24 h before the test.

First, the participants performed a 30 s isokinetic sprint test on a cycle ergometer with subsequent measurements of whole-blood lactate concentration for the determination of VLa_max_. After a 60 min break, a graded exercise test on a treadmill (second test) was performed to determine VO_2max_ and running velocity at the onset of blood lactate accumulation (OBLA; 4 mmol∙L^−1^) [4] and at the modified maximal deviation point (mDmax) [24]. The cLT_An_ was determined according to the calculation scheme described by Mader and Heck [3], as well as by Hauser et al. [22], and subsequently compared with OBLA and mDmax. To evaluate the validity of cLT_An_, OBLA, and mDmax as indicators of endurance performance, running velocities at each concept were compared with the participant’s performance (average velocity (m·s^−1^)) over various distances (3000 m, 5000 m, and 10,000 m). One participant did not provide performance data, so only data from nine participants were included in correlation analysis.

### 2.3. Isokinetic Sprint Test and VLa_max_ Determination (Performance Capacity of Glycolysis)

The participants first performed a 10 min standardized warm-up at 1.5 W kg^−1^ body mass. After an additional passive rest for 5 min, a 30 s sprint test was performed on a cycle ergometer adjusted to an isokinetic mode set to a cadence of 120 rpm [25,26]. Participants were instructed to perform the test in a sitting position and were verbally encouraged throughout the test to achieve and maintain maximal effort. After the sprint, participants took a rest in a sitting position for 10 min. Immediately before sprint testing, as well as every minute after the sprint bout (1′–10′), 20 μL of capillary blood was taken from the earlobe for lactate analysis (Biosen C-line; EKF Diagnostic Sales, Magdeburg, Germany). The VLa_max_ was calculated using the following equation [27]:

VLa_max_ (mmol L^−1^ s^−1^) = ([La]_peak_ − [La]_rest_) ∙ (*t*_exerc_ − *t*_alac_)^−1^ (1)

where La_peak_ (mmol L^−1^) is the peak post-exercise lactate concentration, La_rest_ (mmol L^−1^) is the resting lactate concentration, *t*_exerc_ (s) is the duration of exercise, and *t*_alac_ (s) is the period at the beginning of exercise in which no lactate formation is assumed. According to Heck et al. [27], *t*_alac_ was set to 5.5 s for all participants.

### 2.4. Graded Exercise Running Test and VO_2max_ and LT_An_ Determination

The graded exercise test was performed on a treadmill (Woodway, Weil am Rhein, Germany), which started at 2.4 m s^−1^ and increased by 0.4 m s^−1^ every 5 min until volitional exhaustion was reached. After each step of the graded exercise test, a 30 s rest was given for blood sampling. Furthermore, heart rate (HR) (H7, Polar Electro Oy, Kempele, Finland) and breath-by-breath expired gases (Cortex Metalyzer II, Leipzig, Germany) were continuously measured throughout the test. The VO_2max_ corresponded to the highest value measured (moving average of 30 s) during the test.

Blood lactate concentrations during the incremental tests were plotted against running velocity and then fitted by a third-order polynomial function. Running velocity at OBLA was set as the point at which blood lactate concentration reached 4 mmol∙L^−1^ [4]. mDmax was identified as the point on the third-order polynomial curve that yielded the maximal perpendicular distance to a straight line formed by the peak lactate point, and by the point of the first rise in blood lactate concentration at which the slope of the fitted lactate curve was equal to 1.00 [24].

### 2.5. Calculation of Running Velocity at cLT_An_

To determine cLT_An_, the oxidative and glycolytic energy production depending on exercise intensity must initially be known, which can be expressed as the activity of oxidative phosphorylation (VO_2ss_) and glycolysis (VLa_ss_), respectively [3]. The theoretical background of the applied equations and constants is explained in detail by previous publications [3,22].

**Figure 2:**
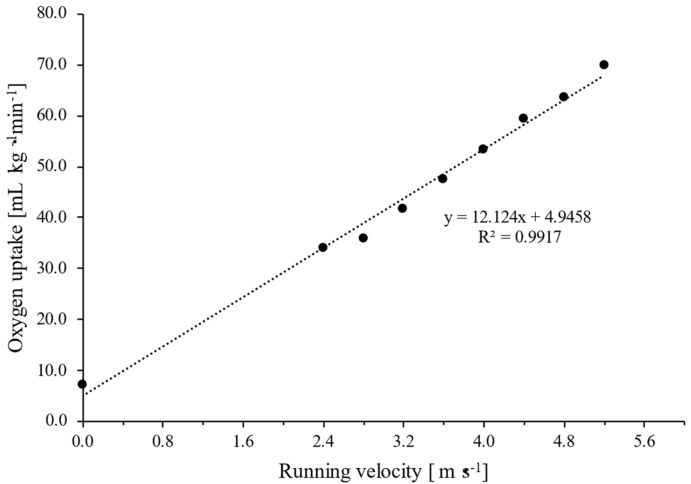
An exemplary description of the determination of the individual *Ks*4 (constant value of the relationship between oxygen demand and running velocity). The slope of the regression line corresponds to *Ks*4. From this equation, *Ks*4 for this runner is 12.1 mL·kg^−1^·min^−1^ per 1 m·s^−1^.

According to Mader and Heck [3], the implementation of the metabolic simulation model requires knowing the free ADP concentration, which is the main regulating substrate for the activation of VO_2ss_ and VLa_ss_. Since there is no simple and practical procedure for measuring free ADP concentration, the ADP-dependent equations in the previous study were transposed into VO_2ss_-dependent equations [22]. On this occasion, the term “VO_2ss_” represents the steady-state oxygen consumption at a constant work rate [3,22]. Hauser et al. [22] calculated the VO_2ss_ in relation to exercise intensity based on the assumption of a linear relationship between oxygen demand (VO_2_) and workload. Thereby, a constant value for VO_2_ per 1 W (*Ks*4 = 11.7 mL O_2_ W^−1^) was used for all participants based on the data of previous cycling experiments [3,28]. However, it should be noted that VO_2_ in running is more affected by an athlete’s exercise economy (i.e., metabolic cost at a given workload) than in cycling. Running economy was shown to be influenced by several physiological and biomechanical factors [29], which can lead to greater inter-individual variation in comparison to the cycling economy due to weight-bearing activity [30]. Therefore, it is necessary to determine *Ks*4 (mL kg^−1^·min^−1^ per 1 m∙s^−1^ running velocity) individually, by plotting VO_2_ during incremental tests against running velocity. The *Ks*4 corresponded to the slope of linear regression (y = mx + b) between VO_2_ and running speed (Figure 2).

After determining the individual *Ks*4, the VO_2ss_ in relation to running velocity was calculated with Equation (2).

VO_2ss_ (mL kg^−1^ min^−1^) = *V Ks*4 + VO_2rest_ (2)

where v (m s^−1^) is the running velocity, VO_2rest_ (mL kg^−1^ min^−1^) is the resting oxygen uptake, and *Ks*4 is the constant value of the relationship between oxygen demand and the running velocity (i.e., mL kg^−1^ per 1 m s^−1^ running velocity).

By knowing VO_2ss_ (from resting level to VO_2max_), it is possible to calculate VLa_ss_ (lactate formation) as a function of VO_2ss_, as demonstrated in the following equation:

(3) $${\mathrm{VLa}}_{\mathrm{ss}}\;(\mathrm{mmol}\;L^{-1}\;\min^{-1})=\frac{60\;\cdot \;\dot{V}Lamax}{1+\left(\frac{\mathrm{Ks}2}{{\sqrt{\frac{\mathrm{Ks}1\cdot {\dot{V}O}_{2\mathrm{ss}}}{{\dot{V}O}_{2\max}-{\dot{V}O}_{2\mathrm{ss}}}}}^{3}}\right)}$$

where VLa_max_ (mmol L^−1^·s^−1^) is the maximal glycolytic rate, VO_2max_ (mL kg^−1^ min^−1^) is the maximal oxygen uptake, VO_2ss_ (mL kg^−1^ min^−1^) is the steady-state oxygen consumption, and Ks1 and Ks2 are the 50% activity rate constant of oxidative phosphorylation (0.0631) and glycolysis (1.331), respectively [22].

Furthermore, the maximal lactate elimination rate (VLa_oxmax_) which depends on VO_2ss_ can also be calculated based on the experimentally estimated value of lactate equivalent (i.e., the amount of oxidized lactate per unit O_2_), lactate distribution volume [3], and using the following equation:

(4) $${\mathrm{VLa}}_{\mathrm{oxmax}}\;(\mathrm{mmol}\;L^{-1}\;\min^{-1})=\frac{\mathrm{lactate-equivalent}}{\mathrm{lactate}\;\mathrm{distribution}\;\mathrm{volume}}\;\cdot {\mathrm{VO}}_{2ss}=\frac{0.02049}{0.4}\cdot {\mathrm{VO}}_{2ss}$$

where VLa_oxmax_ (mmol L^−1^ min^−1^) is the maximal lactate elimination rate as a function of the steady-state oxygen consumption (VO_2ss_; mL kg^−1^ min^−1^) [22].

According to Hauser et al. [22], lactate-equivalent and lactate distribution volume were set to 0.02049 mmol lactate per 1 mL O_2_ and 0.4 L H_2_O per kg body weight, respectively. Thus, simulating the simultaneous lactate formation and elimination depending on the metabolic rate or running speed can be carried out based on the individual VO_2max_ and VLa_max_ value, as well as body weight. cLT_An_ is defined as the running velocity at which the lactate formation is exactly equal to elimination (i.e., VLa_ss_ = VLa_oxmax_).

### 2.6. Statistical Analysis

For statistical analysis of the data, the software IBM SPSS version 24 (Chicago, IL, USA) was used. Descriptive statistics of the data are presented as means ± standard deviation (±SD). The normal distribution and the variance homogeneity were verified using the Shapiro–Wilk test and Mauchly test of sphericity, respectively. Statistically relevant differences between the three LT_An_ concepts were determined using one-way repeated measure ANOVA with Bonferroni correction for post hoc tests. Statistical differences were considered to be significant for *p* ≤ 0.05. To estimate the practical relevance, effect sizes (partial eta squared, *η_p_²*) were calculated for the main effect. According to Cohen [31], a *η_p_²* ≥ 0.01 indicates small effects, ≥0.059 medium effects, and ≥0.138 large effects. To display the concordance between the LT_An_ concepts, Bland–Altman plots were constructed. Furthermore, the intra-class correlation coefficients (ICC) were calculated based on a single-measure two-way mixed-effects model. For evaluating the degree of agreement between cLT_An_ vs. OBLA or mDmax, the “absolute agreement” type of analysis (ICC (2,1)) was chosen. For the comparison of each LT_An_ concept vs. 3000 m, 5000 m, or 10,000 m, we chose the “consistency” type of analysis (ICC (3,1)). According to Koo and Li [32], the degree of agreement was interpreted as follows: <0.50 = poor, 0.50–0.75 = moderate, 0.75–0.90 = good, and >0.90 = excellent. Pearson’s correlations were also calculated and interpreted as follows: 0.0–0.3 = negligible, 0.3–0.5 = low, 0.5–0.7 = moderate, 0.7–0.9 = high, and 0.9–1.0 = very high [33].

## 3. Results

Individual values of maximal metabolic performance tests and individual running velocities at each LT_An_ concept are presented in Table 1.

Repeated measures ANOVA showed a significant difference between LT_An_ concepts with a large effect (*p* < 0.01, *η*^2^*_p_* = 0.63). Post hoc analysis using Bonferroni correction revealed that running velocity at OBLA was significantly higher compared to cLT_An_ and mDmax (*p* < 0.01). No significant difference was found between running velocity at cLT_An_ and mDmax (*p* = 0.49). The cLT_An_ indicated a high correlation with OBLA (*r* = 0.83, *r*^2^ = 0.70, *p* < 0.01) and mDmax (*r* = 0.81, *r*^2^ = 0.65, *p* < 0.01). Between OBLA and mDmax, there was a very high correlation (*r* = 0.94, *r*^2^ = 0.89, *p* < 0.001).

**Figure 3:**
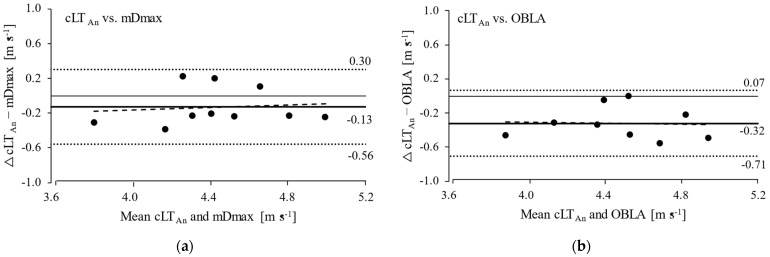
Bland–Altman Plots: differences in running velocity at calculated anaerobic lactate threshold (cLT_An_) vs. modified maximal deviation method (mDmax; (**a**)) and onset of blood lactate accumulation (OBLA; (**b**)). The solid lines indicate the mean difference; the dotted lines indicate the limits of agreement (mean ± 1.96 SD); the dashed lines represent the fitted linear regression.

According to the Bland–Altman Plots (Figure 3), the mean difference in cLT_An_ was −0.13 ± 0.43 m∙s^−1^ and −0.32 ± 0.39 m∙s^−1^ compared to mDmax and OBLA, respectively. The intraclass correlation coefficient comparing cLT_An_ with mDmax showed a good agreement (ICC = 0.87), whereas a moderate agreement was shown between cLT_An_ and OBLA (ICC = 0.74).

**Figure 4:**
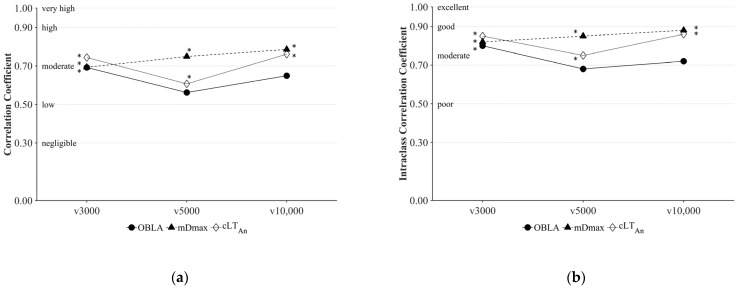
Correlations (**a**) and intraclass correlation coefficients (**b**) of the running velocity at onset of blood lactate accumulation (OBLA), modified maximal deviation method (mDmax), and calculated anaerobic lactate threshold (cLT_An_) compared to average running velocity over 3000 m (v3000), 5000 m (v5000), and 10,000 m (v10,000); * *p* < 0.05.

The mean running velocities over the distances of 3000 m, 5000 m, and 10,000 m were 5.65 ± 0.29 m s^−1^, 5.37 ± 0.26 m s^−1^, and 5.03 ± 0.26 m s^−1^, respectively. cLT_An_ and mDmax indicated moderate to high correlations with running performance over all distances observed (cLT_An_: 0.61 < *r* < 0.76, 0.37 < *r*^2^ < 0.58, *p* < 0.05; mDmax: 0.69 < *r* < 0.79, 0.48 < *r*^2^ < 0.62, *p* < 0.05), whereby OBLA had the poorest correlations (0.56 < *r* < 0.69, 0.32 < *r*^2^ < 0.48, *p* ≤ 0.09) compared to other concepts in most cases (Figure 4a). The intraclass correlation (Figure 4b) also revealed good concordance of cLT_An_ (ICC = 0.75–0.86) and mDmax (ICC = 0.82–0.88) with running performance over all distances observed, whereas OBLA showed only moderate concordance (ICC = 0.68–0.80) in most cases.

## 4. Discussion

The purpose of this study was to determine cLT_An_ in running by adapting the mathematical model for metabolic simulation previously described by Mader and Heck [3] and Hauser et al. [22]. cLT_An_ demonstrated moderate to good concordance with the established concepts in determining the running velocity at LT_An_. Although cLT_An_ provided lower running velocity compared to mDmax and OBLA, the correlation of cLT_An_ with the endurance running performance was similar compared to mDmax and even better compared to OBLA.

One of the relevant criteria for the practical application of a laboratory test parameter is its relationship with competitive performance. A comprehensive review by Faude et al. [8] demonstrated moderate to high correlations (*r* = 0.66–0.92) between various LT_An_ concepts and performance in endurance running competitions and therefore justified the practical application of those concepts in sports diagnostics. Even though cLT_An_ did not indicate significantly superior results, its good concordance (ICC = 0.75–0.86) with mDmax and OBLA, as well as comparable correlations (*r =* 0.61–0.76) with competition performance, can support its applicability as a valid indicator to assess an athlete’s endurance performance.

The metabolic simulation model (cLT_An_) incorporates the influence of individual VO_2max_, VLa_max_, and *Ks*4 on LT_An_, as well as their combined effects [18,22]. This could enable a more differentiated approach in the interpretation of the endurance performance of an athlete. The individually determined *Ks*4 values are dependent on individual exercise economy, expressed by the relationship between energy demand and running velocity [29]. Especially in well-trained athletes with similar VO_2max_, running economy has been shown to be a crucial indicator of distance running performance [29,34,35]. The consideration of individual physiological profiles allows specific explanations of how equal and/or different endurance performance can be achieved regarding the interplay of single metabolic parameters [18]. For instance, participants 3 and 7 in our study showed similar aerobic and anaerobic capacities (VO_2max_: 80.3 vs. 80.1 mL kg^−1^∙min^−1^, VLa_max_: 0.33 vs. 0.31 mmol L^−1^ s^−1^); however, participant 3 displayed a much higher speed at LT_An_ regardless of the used LT_An_ concept (Table 1) and, consequently, better performance compared to participant 7 (e.g., 10,000 m running time: 30 vs. 32 min). In this case, the performance differences could be explained by much lower *Ks*4 (13.2 vs. 14.2 mL kg^−1^ min^−1^ per 1 m s^−1^). A recent training study used the metabolic simulation-model-detected training-induced changes in single performance capacities (i.e., VO_2max_ and VLa_max_). The authors reported specific explanations of changes in endurance performance (MLSS) [36], which highlights the potential for the practical application of the model.

Despite the moderate to good agreement with other LT_An_ concepts, cLT_An_ systematically provides lower running velocities in our study (Figure 3). This discrepancy could be attributed to the underrated VO_2max_ by using a graded exercise test. The main reason we used a graded incremental protocol, instead of a ramp protocol, was to concurrently determine OBLA and mDmax, as well as the relationship between steady-state oxygen demand and running velocity (i.e., individual *Ks*4). However, the mean time to exhaustion of our test protocol was ~38 min, which is significantly longer than the “optimal” test duration for assessing VO_2max,_ as suggested by previous studies [37,38,39]. Sperlich et al. [40] reported that VO_2max,_ achieved with the same graded exercise test protocol as in our study, was significantly lower (on average 2 mL min^−1^ kg^−1^) than assessed by incremental tests with shorter test duration (ranged from 7–11 min). Hauser [28] showed that a theoretical 25% increase in VO_2max_ (and constant VLa_max_, and *Ks*4) leads to a 44% increase in calculated MLSS in cycling. Indeed, cLT_An_ is increased by ~0.2 m s^−1^ when the participant’s VO_2max_ is increased by 2 mL min^−1^ kg^−1^ (and constant values of VLa_max_, and *Ks*4), and thus the difference between running speed at cLT_An_ and the other LT_An_ is reduced (data not presented). To solve the underestimation of VO_2max_, further work should use a VO_2max_ verification bout [41,42] or a combined step- and ramp-exercise protocol [43]. Such protocols could ensure the appropriate determination of VO_2max_ and the individual *Ks*4 at the same time, as two core parameters of the metabolic simulation model.

Another potential contributing factor to the difference between cLT_An_ and other LT_An_ concepts could be the run-nonspecific test procedure for the assessment of VLa_max_ and its influence on cLT_An_. The cycling sprint test is an established anaerobic test for nearly all sports disciplines. Thus, we determined the participant’s VLa_max_ using an isokinetic cycle sprint [22,23,36,44]. However, the peak post-exercise lactate concentration, which is a key parameter for the estimation of VLa_max_, is dependent on the exercise modality used in tests [44]. Unfortunately, up to now no established running-specific test procedure for VLa_max_ determination exists. Just recently, Quittmann et al. [45] attempted to measure VLa_max_ and sprint performance parameters using a running sprint test. However, this study used fixed distances, rather than a fixed time for the sprint test, which might influence VLa_max_ determination. Whether and how VLa_max_ estimation and cLT_An_ determination would be affected by applying a running-specific anaerobic test procedure remain to be clarified.

Since VO_2ss_ contributes as a core parameter to the calculation of both the lactate formation and elimination rate at any given running velocity, it is necessary to determine VO_2ss_ (from resting level to VO_2max_) as precisely as possible. For the determination of VO_2ss_, the relationship between oxygen demand and running velocity (*Ks*4) plays an important role [3]. In contrast to the previous study in cycling [22], we individually determined the *Ks*4 value considering the inter-individual variation in the running economy. Typically, it is assumed that there is a linear relationship between VO_2_ and workload. This has been supported by several investigations indicating a nearly invariant oxygen cost of transport (calculated by dividing oxygen uptake by running velocity, mL kg^−1^ km^−1^) over a range of running speeds (2.0–4.0 m s^−1^) [46,47]. However, these studies investigated the individual running energetics only from the start of exercise until LT_An_ intensity and not till exhaustion. Daniels and Daniels [48] suggested that the metabolic demand of running is not exclusively dependent on running speed and can vary with an athlete’s specialized background. They found that most of the 800–1500 m specialists in their study showed an equal oxygen cost of transport over all intensities examined. In contrast to that, the specialists in longer distances (3000 m—marathon) mostly showed an increased oxygen cost of transport at exercise intensities above 70% of VO_2max_ [48]. These findings emphasize the importance of considering the individual running energetics over all possible test speeds to assess the performance difference between athletes. To what extent the running energetics, especially near the LT_An_ intensity, differ between athletes, and how they affect the LT_An_, is unclear. With respect to the previous model in cycling [22], we, therefore, decided to use the *Ks*4 from a linear fit to calculate VO_2ss_ in our study. However, there is abundant space for further progress in analyzing the relationship between metabolic rate and running velocity and its influence on cLT_An_ determination. For instance, a curvilinear fit suggested by Batliner et al. [49] might better assess the inter-individual difference in running energetics, especially around and above the LT_An_ intensity, which might consequently lead to an improved performance prediction of cLT_An_.

In addition to the above methodological limitations, it is important to note that our data did not address the basic variability and reproducibility of each physiological measure (VO_2max_, VLa_max_, and *Ks*4), which are also relevant quality criteria for the application of the cLT_An_. However, previous research in cycling already demonstrated a very high reliability for both VO_2max_ and VLa_max_, as well as the calculated MLSS from these two parameters [23]. Further studies with a longitudinal analysis in running should be carried out to investigate the reliability and sensitivity of the single performance tests and metabolic simulation model for detecting performance changes.

## 5. Practical Applications

The present study suggests that the mathematical model for metabolic simulation could be applied to assess an athlete’s endurance performance in running by considering multiple physiological parameters. Considering multiple physiological measures, the metabolic simulation model (cLT_An_) provides an insight into the complex interplay of single metabolic systems and their influence on endurance performance. This allows a differentiated interpretation of the athlete’s performance, which could be useful for establishing training interventions targeting and eliminating specific weaknesses in the physiological profile of an athlete.

## 6. Conclusions

The metabolic simulation model considers different metabolic parameters to evaluate an athlete’s performance profile. In determining running velocity at LT_An_, the metabolic simulation model (cLT_An_) revealed a moderate to good agreement with other established concepts. However, the velocity at cLT_An_ was lower with regard to the other LT_An_ concepts. With regard to the compared LT_An_ concepts, comparable and partially better correlations between cLTan and the endurance performance of sub-elite middle- and long-distance runners were found.

## Figures and Tables

**Table 1:** Body mass, maximal oxygen uptake (VO_2max_), maximal lactate production rate (VLa_max_), constant value of the relationship between oxygen demand and running velocity (*Ks*4), and running velocity at the onset of blood lactate accumulation (OBLA), at the modified maximal deviation method (mDmax) and the calculated anaerobic lactate threshold (cLT_An_) for each participant.

Participant	Body Mass (kg)	VO_2max_ (mL kg^−1^∙min^−1^)	VLa_max_ (mmol L^−1^ s^−1^)	*Ks*4 (mL kg^−1^∙min^−1^ per 1 m s^−1^)	OBLA (m s^−1^)	mDmax (m s^−1^)	cLT_An_ (m s^−1^)
1	59.2	74.6	0.38	12.1	5.19	4.93	4.70
2	64.4	70.0	0.32	12.1	4.97	4.65	4.42
3	64.4	80.3	0.33	13.2	-	5.12	4.87
4	72.6	68.0	0.33	11.8	4.43	4.16	4.38
5	72.3	65.4	0.42	11.3	4.54	4.43	4.20
6	68.5	62.3	0.46	10.7	4.31	4.37	3.99
7	59.1	80.1	0.31	14.2	4.53	4.33	4.53
8	73.2	67.4	0.55	10.8	4.77	4.52	4.31
9	58.4	68.7	0.33	11.0	4.94	4.61	4.72
10	65.4	60.8	0.50	11.0	4.13	3.97	3.66
Mean ± SD	65.8 ± 5.8	69.8 ± 6.7	0.39 ± 0.09	11.8 ± 1.1	4.65 ± 0.35	4.44 ± 0.28 *	4.32 ± 0.34 *

* significantly different compared to OBLA (*p* < 0.01).

## Data Availability

The data presented in this study are available on request from the corresponding author.
